# Laser interferometric investigation of solute transport through membrane-concentration boundary layer system

**DOI:** 10.1007/s10867-015-9387-y

**Published:** 2015-06-24

**Authors:** Sławomir Wąsik, Arkadiusz Bryll, Marcin Drabik, Kazimierz Dworecki, Andrzej Ślęzak

**Affiliations:** Institute of Physics, Jan Kochanowski University, Świętokrzyska 15, 25406 Kielce, Poland; Department of Biophysics, Częstochowa University of Technology, 36B Armia Krajowa Al., 42200 Częstochowa, Poland

**Keywords:** Membrane transport, Solute permeability coefficient, Concentration boundary layers, Concentration polarization, Laser interferometry

## Abstract

We investigate diffusive transport in a membrane system with a horizontally mounted membrane under concentration polarization conditions performed by a laser interferometry method. The data obtained from two different theoretical models are compared to the experimental results of the substance flux. In the first model, the membrane is considered as infinitely thin, while in the second one as a wall of finite thickness. The theoretical calculations show sufficient correspondence with the experimental results. On the basis of interferometric measurements, the relative permeability coefficient (*ζ*_s_) for the system, consisting of the membrane and concentration boundary layers, was also obtained. This coefficient reflects the concentration polarization of the membrane system. The obtained results indicate that the coefficient *ζ*_s_ of the membrane-concentration boundary layer system decreases in time and seems to be independent of the initial concentration of the solute.

## Introduction

The concentration boundary layers (CBLs) created near membrane surfaces act as pseudo-membranes in series with the physical membrane. Thus, the permeability coefficient of the system, consisting of membrane-concentration boundary layers (*ω*_s_), is smaller than the permeability coefficient of the membrane (*ω*_m_) [1]. A previous investigation using a chamber system showed that the flux of the dissolved substance *J*_s_ in a gravitationally unstable configuration of a membrane system (when the CBLs are destroyed by gravitational convection) in a stationary state is approximately nine times higher than in a gravitationally stable configuration. If the stirring is strong enough, the CBL thickness is minimized and then both gravitational configurations of the system are equal [2].

The concentration boundary layers and the concentration polarization phenomenon strongly influence the transport processes in artificial as well as in biological systems [3–12]. They play an important role in many physiological processes such as coupling of water transport to active solute transport in lateral intercellular spaces of epithelia, or the conservation of solutes transiently leaving a cell during an action potential [3, 13–16]. The rate and effectiveness of chemical transformations within the CBLs are affected by the availability of reactants. Boundary layers near a membrane are the source of an inaccurate Michaelis constant in the membrane transport [17]. It has also been suggested that the thickness of CBLs has a regulative function for intestinal absorption [6, 14]. Changes in the epithelial function or luminal stirring can, for example, readily influence the absorption of small molecules [13, 14, 18, 19]. In many interesting cases, diffusion of solutes through CBLs is accompanied by chemical reactions [20].

The concentration polarization phenomenon is particularly observed under microgravity conditions [21]. According to the model developed by Schatz and coworkers, the mechanism of action of the gravity force on a cell consists of inducing adaptation provoked by physicochemical changes in the cellular environment. A lack of natural convection in microgravity favors the formation of diffusion layers around the cells. In these cells, the uptake rate of oxygen and nutrients is changed and therefore the cell metabolism is significantly affected. The cells in a microgravity field show major changes, involving metabolism, cytoskeleton, membrane structure, gene regulation, shape, and many other biological properties [22–27].

The theoretical modeling of CBLs creation/destruction (concentration polarization phenomenon) is based, among others, on the Nernst–Planck, Poisson, Stokes, Fick, and/or Kedem–Katchalsky [5, 7, 9–12, 28–32] equations. To describe the concentration polarization of a system, we introduce the relative permeability coefficient (*ζ*_s_) for a system consisting of membrane and concentration boundary layers [1], also called the diffusive Katchalsky factor [33, 34] and propose the model equation for this coefficient and its dependence on different parameters [2].

The coefficient *ζ*_*s*_ is defined as [2]:

1 $$ \begin{array}{cc}\hfill {\zeta}_s={J}_s{\left({J}_s^0\right)}^{-1}={\omega}_s{\omega}_m^{-1}\hfill & \hfill \left(0\le {\zeta}_s\le 1\right)\hfill \end{array} $$

where *J*_s_ and *J*_s_^0^ are the substance fluxes obtained under the polarization and well-stirred conditions, respectively. On the basis of the modified Kedem–Katchalsky model the following formula is provided [1, 2]:

2 $$ {\zeta}_s={\left[1+RT{\omega}_m\left(\frac{\delta_1}{D_1}+\frac{\delta_2}{D_2}\right)\right]}^{-1}, $$

where *R* is the gas constant, *T* is the thermodynamic temperature, *δ*_1_ and *δ*_2_ the concentration boundary layer thicknesses, and *D*_1_ and *D*_2_ are the diffusion coefficients in the *δ*_1_ and *δ*_2_ layers, respectively.

After appropriate assumptions that *δ*_1_ = *δ*_2_ = *δ* and *D*_1_ = *D*_2_ = *D*, formula (2) can be written as:

3 $$ {\zeta}_s={\left(1+\frac{2RT{\omega}_m\delta }{D}\right)}^{-1}. $$

We present the experimental results for the substance flux and coefficient *ζ*_s_ obtained interferometrically in a membrane system under concentration polarization conditions. Most of the work devoted to this issue applies to stationary states of the system. The laser interferometry method is accurate and a precise tool to comprehensively study the transport phenomena, quantitatively and qualitatively, in gels, liquids and membranes in stationary as well as non-stationary states. This method has been used by our team and other researchers [35–40] in studies of artificial and biological systems.

This paper also includes a comparison of the results with the data calculated from two different theoretical models as well as from the formula for coefficient *ζ*_s_ obtained from a modified Kedem–Katchalsky model. The laser interferometry method is a convenient technique for testing the theoretical models of substance transport and allows accurate determination of the membrane parameters on the basis of these models. It should be stressed that an important result of our study in addition to determining the relative permeability coefficient for the membrane/layers system is also to determine the transport parameters of the membrane, i.e., the selectivity coefficient (γ) for an infinitely thin membrane and the partition coefficient (*k*) of the substance in the membrane/solution interface for the membrane of the finite thickness. The knowledge of these parameter values allows for a better understanding of the structure and permeability of the membrane, particularly in the study of biological membrane models [41, 42].

## Materials and methods

### Theoretical description

The first model treats the membrane as infinitely thin. The initial condition for this model is:

4 $$ C\left(x,t=0\right)=\left\{\begin{array}{cc}\hfill {C}_h={C}_0\hfill & \hfill \mathrm{f}\mathrm{o}\mathrm{r}\ x<0\hfill \\ {}\hfill {C}_l=0\hfill & \hfill \mathrm{f}\mathrm{o}\mathrm{r}\ x>0\hfill \end{array}\right\} $$

The concentration profile that describes the spatio-temporal concentration distribution inside the layers for this model is [43]:

5 $$ {C}_l\left(x,t\right)={C}_0\frac{1-\gamma }{2} erfc\left(\frac{x}{2\sqrt{Dt}}\right), $$

where *γ* is the diffusive selectivity of the membrane for the solute.

The second model treats the membrane as a wall of finite thickness *l*. The initial boundary conditions for this model are:

6 $$ C\left(x,t=0\right)=\left\{\begin{array}{lll}{C}_h\left(x,0\right)={C}_0\hfill & \mathrm{f}\mathrm{o}\mathrm{r}\hfill & -\infty <x<0\hfill \\ {}{C}_m\left(x,0\right)=-k{C}_0\left(\raisebox{1ex}{$x$}\!\left/ \!\raisebox{-1ex}{$l$}\right.+1\right)\hfill & \mathrm{f}\mathrm{o}\mathrm{r}\hfill & 0<x<l\hfill \\ {}{C}_l\left(x,0\right)=0\hfill & \mathrm{f}\mathrm{o}\mathrm{r}\hfill & l<x<\infty \hfill \end{array}\right\} $$

where *C*_h_, *C*_l_ denote higher and lower concentrations respectively, *C*_*m*_ is the concentration of the solute inside the membrane and *k* is the solute partition coefficient.

In accordance with Ref. [44], the concentration profile is:

7 $$ {C}_l\left(x,t\right)={C}_0\left[\frac{2r{\left({D}_mt\right)}^{1/2}}{\left(r+1\right)l}\times ierfc\left(\frac{x-l}{2{(Dt)}^{1/2}}\right)\right.+\frac{4{r}^2{\left({D}_mt\right)}^{1/2}}{\left({r}^2-1\right)l}{\displaystyle \sum_{n=1}^{\infty }{\left(\frac{1-r}{1+r}\right)}^n}\times \left. ierfc\left(\frac{nl}{2{\left({D}_mt\right)}^{1/2}}+\frac{\left(x-l\right)}{2{(Dt)}^{1/2}}\right)\right], $$

where $r=k\sqrt{D_m/D}$ and *D*_m_ is the solute diffusion coefficient in the membrane.

### Membrane system

**Fig. 1:**
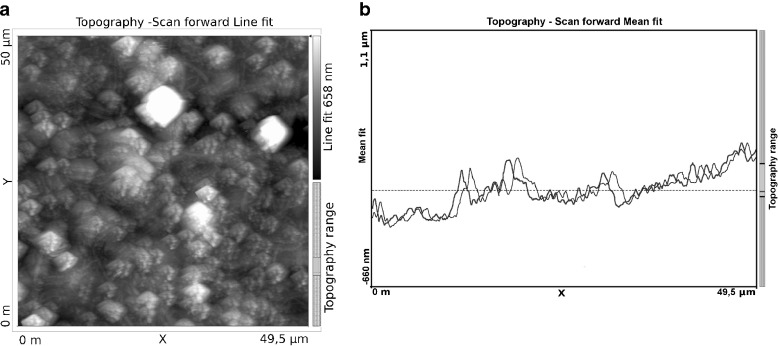
Atomic force microscopy topographic image of the Nephrophane membrane surface (**a**) and an altitude profile of the Nephrophane membrane (**b**)

**Fig. 2:**
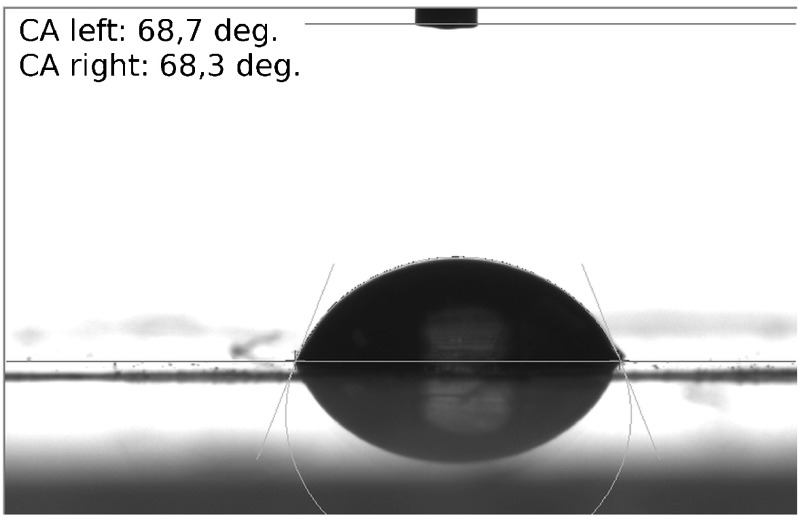
Contact angle measurement of water drops for the Nephrophane membrane

**Fig. 3:**
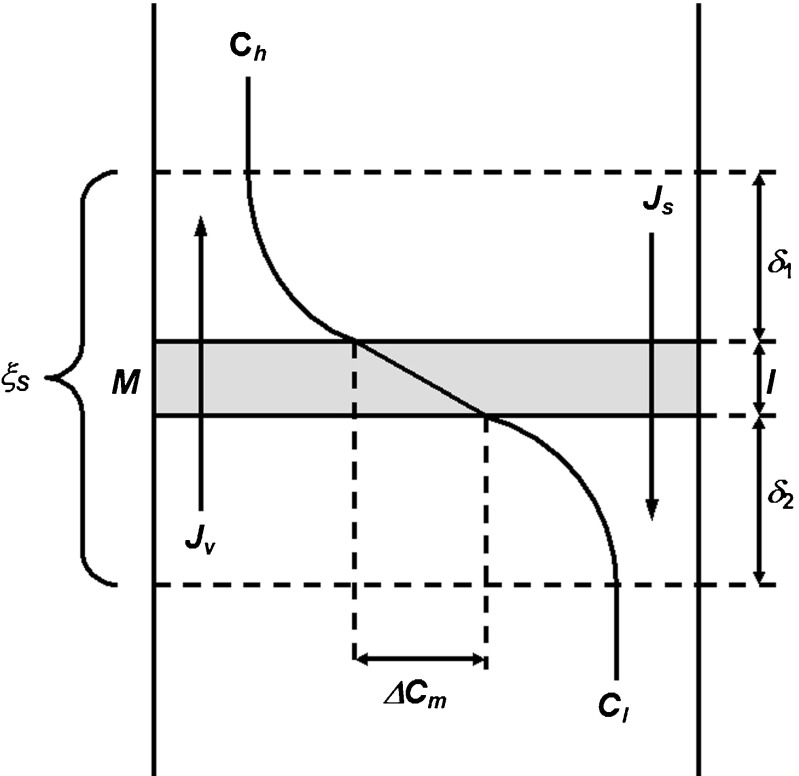
Membrane system under concentration polarization conditions

The membrane system under study consists of two glass vessels with high homogeneity (internal dimensions 70-mm height, 10-mm width, and 7-mm optical path length) separated by a horizontally mounted Nephrophane hemodialyzer membrane after partial hydration. Nephrophane is a microporous membrane made from cellulose acetate ([trio-acetate cel-(O-CO-CH_3_)_n_]) of a spongy structure. The structure of the membrane surface is illustrated by an atomic force microscope (AFM). The measurement was performed in tapping mode with a probe Tap190Al-G (BudgetSensors, Bulgaria). Figure 1a shows the membrane surface topography and Fig. 1b shows a randomly selected altitude profile of the membrane area indicated in Fig. 1a using a triangle. The two lines illustrate the result of the measurement that was carried out twice in the direction of the* x*-axis (from left to right and from right to left). The surface roughness was determined based on measurements done with the AFM technique. The root mean square (RMS) roughness value is 0.1623 μm. The calculations were performed using WSxM 5.0 Develop 6.5 software [45]. Measurements with water drops (5μl) indicate that the contact angle is about 68° and reveal the hydrophilic nature of the membrane (Fig. 2), which was measured using goniometer OCA 15EC (Dataphysics, Germany). The permeability coefficient of the Nephrophane membrane for ethanol is equal to *ω*_m_ = 1.43 × 10^−9^ mol/Ns. The upper compartment of the membrane system was filled with ethanol solutions of concentrations *C*_h_ (*C*_h_ = 125, 250, 500, and 750 mol/m^3^) whereas in the lower one it was pure water (*C*_l_ = 0). In such a configuration of the system, the water and dissolved substance diffusing across the membrane will lead to the formation of CBLs that cause the concentration polarization of the membrane (Fig. 3). Under concentration polarization conditions, the concentrations of solutions at the membrane-layer surfaces are different from concentrations in the bulk.

### Experimental setup

Studies of the flux *J*_s_ and the coefficient *ζ*_s_ were carried out using the method of laser interferometry. The experimental setup and the measurement method were described in our previous papers [46, 47]. The computerized data acquisition system measures the deviation *d*(*x*,*t*) of these fringes from their straight line run and thus allows to determine the concentration profiles *C*(*x*,*t*), the drops of concentration ∆*C*(*x*,*t*) in the CBL and the CBL thickness (δ).

The CBL thickness *δ* was arbitrarily defined as the distance from the membrane–water interface to the point at which the concentration decreases *K-*fold, i.e.,

8 $$ C\left(t,x=0\right)=KC\left(t,x=\delta \right), $$

with *x* = 0 being the membrane surface position. The arbitrary constant *K* is assumed to be equal to *K* = 12.5; however, it may be any other value satisfying specific application requirements. The thickness *δ* obtained by using this criterion seems to be independent of the initial concentration [43]. The molar flux *J*_s_ of the solute is given by:

9 $$ {J}_s=\frac{\varDelta N(t)}{S\ \varDelta t}, $$

where ∆*N*(*t*) is the amount of solute that diffuses in time interval ∆*t* through the membrane with the area *S* from one compartment into another of the membrane system. *N*(*t*) at any time *t* was calculated by integrating the concentration profile *C*(*x*,*t*) according to:

10 $$ N(t)=S{\displaystyle \underset{0}{\overset{\delta }{\int }}C\left(x,t\right)dx.} $$

On the basis of (9) and (10). we obtain:

11 $$ {J}_s(t)=\frac{{\displaystyle \underset{0}{\overset{\delta }{\int }}{C}_l\left(x,t+\varDelta t\right)dx-{\displaystyle \underset{0}{\overset{\delta }{\int }}{C}_l\left(x,t\right)dx}}}{\varDelta t}, $$

where *C*_*l*_ denotes the concentration profile within the layer *δ*.

Recording the interferograms with a given time step ∆*t*, one can reconstruct the concentration profiles at a different time *t*. The measurements were performed during 40 min with the time interval *Δt* = 120 s at the temperature *T* = 295 K.

Considering the obtained profiles, according to formulas (11) and (1), the time dependencies *J*_s_(*t*) and ζ_s_(*t*) were obtained.

Analogous time dependencies were also obtained on the basis of theoretical concentration profiles for the two models of the membrane.

The fluxes *J*_s_^0^ were obtained by using the diffusion cell under stirred conditions [2].

## Results and discussion

**Fig. 4:**
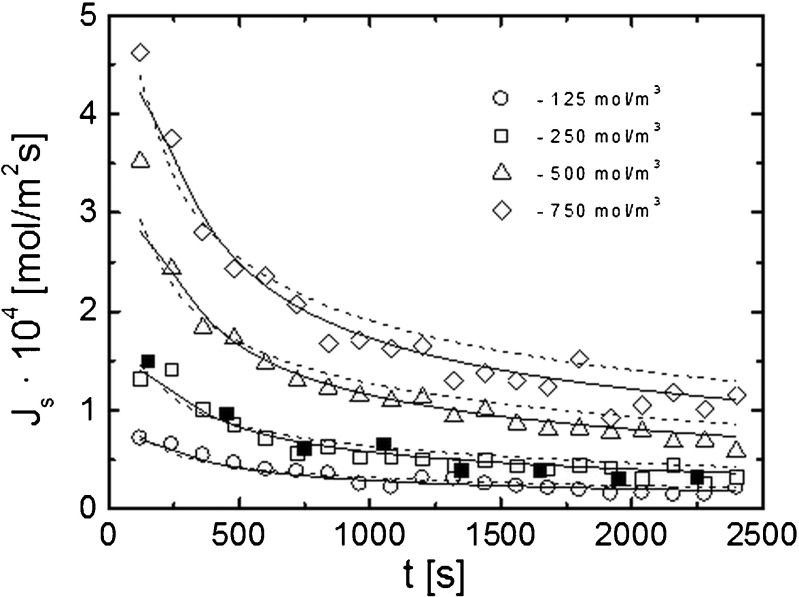
Time characteristics of the diffusive flux (*J*_s_) for different initial concentrations of ethanol solutions.* Open symbols* denote experimental dependencies obtained on the basis of interferometer measurements for concentrations 125, 250, 500, and 750 mol/m^3^; *solid lines* indicate theoretical dependencies obtained for the given concentrations from the first model (5); *dashed lines* are theoretical dependencies obtained from the second model (7); *filled squares* are the fragment of the *J*_s_(*t*) characteristic obtained for a concentration of 250 mol/m^3^ by using the chamber system

**Fig. 5:**
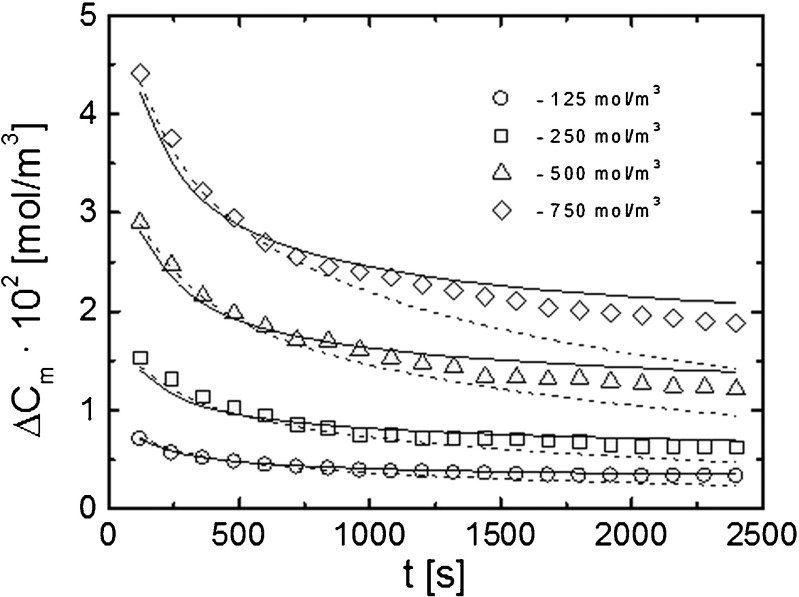
Time dependencies of the concentration difference (∆*C*_m_) on the membrane.* Open symbols* denote experimental dependencies obtained interferometrically; *solid lines* indicate theoretical dependencies obtained from the first model (5); *dashed lines* are theoretical dependencies obtained from the second model (7)

**Fig. 6:**
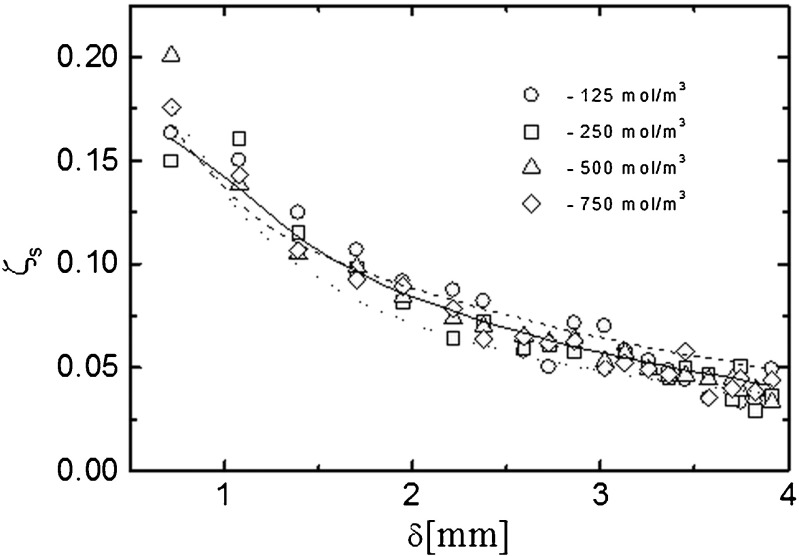
The dependence of the relative permeability coefficient (*ζ*_s_) on CBL thickness (*δ*).* Open symbols* denote the experimental data. *Lines* indicate theoretical dependencies obtained from the first (*solid*) and the second (*dashed*) model. *Dotted line* denotes the dependence obtained according to formula (3) from the time evolution of CBL thickness

**Fig. 7:**
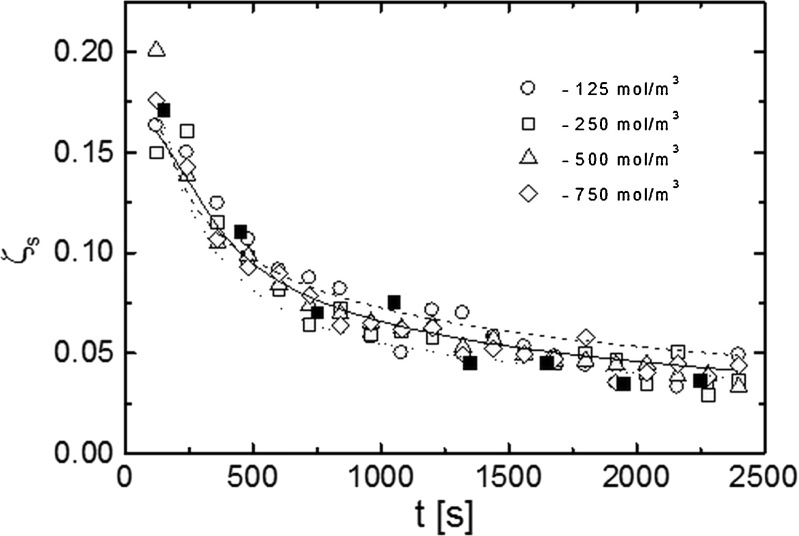
Time characteristics of the relative permeability coefficient (*ζ*_s_) of the M/CBL system.* Open symbols* denote experimental dependencies. *Lines* indicate dependencies obtained from theoretical models;* filled squares* are the fragment of the *J*_s_(*t*) characteristic obtained for a concentration of 250 mol/m^3^ by using the chamber system

**Fig. 8:**
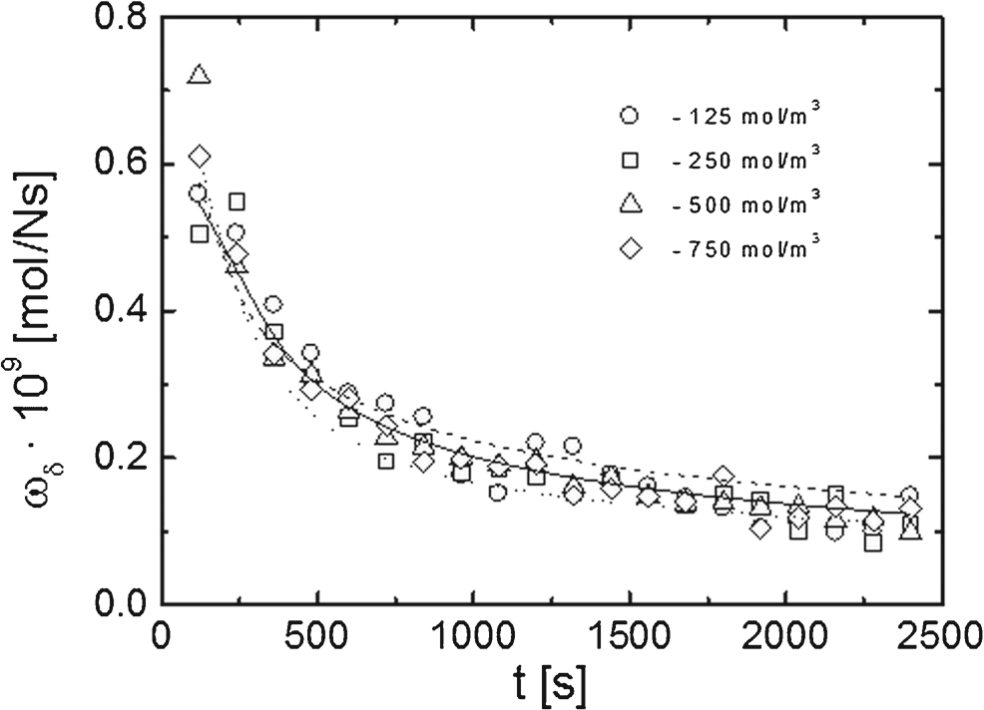
Time dependence of solute layer permeability (ω_*δ*_) calculated on the basis of relative permeability coefficient ζ_s_.* Open symbols* denote the experimental data and the *solid* and *dashed lines* denote theoretical dependencies obtained from the first and second model, respectively. *Dotted line* denotes the dependence obtained according to formulas (3) and (13) from the time evolution of CBL thickness

The experiment results obtained from the computer analysis of the interference images are presented in Figs. 4, 5, 6, 7, and 8. Figure 4 shows a comparison of the time dependencies of the solute flux *J*_s_(*t*) for ethanol solutions with concentrations 125, 250, 500, and 750 mol/m^3^. These dependencies were obtained according to the procedure described in the previous section on the basis of experimental and theoretical concentration profiles.

Taking into account the time dependence of the CBL thickness $\delta (t)=a\sqrt{t}$ (with *a* = 8.1 × 10^−5^ m/s^1/2^) [43] for the first model the following analytical formula was found:

12 $$ {J}_s(t)={C}_0\frac{1-\gamma }{2}\left(\frac{2\left(1- \exp \left(-\frac{a^2}{4D}\right)\sqrt{D}\right)}{\sqrt{\varPi }}+a\  erfc\left(\frac{a}{2\sqrt{D}}\right)\right)\frac{\sqrt{t+\varDelta t}-\sqrt{t}}{\varDelta t}. $$

The diffusion coefficient *D* (*D* = 1.07 × 10^−9^ m^2^/s) was determined from parameter *a* as a fit parameter of time evolution of CBLs. The membrane selectivity γ (γ = 0.20) was determined also as a fit parameter of theoretical concentration profiles (12) to the experimental ones according to the procedure described in Ref. [43].

For the second model, the dependence *J*_s_(*t*) was found on the basis of numerical integration of the concentration profile. The diffusion coefficient in the membrane *D*_m_ (*D*_m_ = 0.56 × 10^−10^ m^2^/s) was determined in a separate experiment using the chamber system under stirring conditions of solutions. The partition coefficient *k* (*k* = 0.25) was obtained as a fit parameter of the theoretical formula (7) to the experimental profiles.

Both theoretical models conform to experiment data for lower concentrations. For higher concentrations and later time points, the experiment and theoretical data show a small discrepancy and the higher values of *J*_s_ are predicted by the second model. The *J*_s_(*t*) characteristic obtained for the concentration of 250 mol/m^3^ by using the chamber system shows a good compatibility with the data obtained from the interferometer measurements.

The plots of the time dependencies of *J*_s_ provide essential information on the membrane transport kinetics. The presented curves indicate that the transition from a non-stationary to a steady state is reached after about 20 min. Visualization and the possibility to observe not only the steady state but also the non-stationary state of transport processes in the system is a great advantage of the laser interferometry method.

The course of changes of the diffusion flux is determined mainly by the concentration on the membrane–solution interface. The CBL formation process leads to a significant reduction in the concentration difference on the membrane, which results in the observed course of changes in the diffusive flux. In Fig. 5, experimental and theoretical (predicted by the models) time dependencies of the concentration difference on the membrane are presented. The discrepancies between the experimental and theoretical values of the concentration difference on the membrane predicted for longer times by the second model may indicate that the partition coefficient *k* varies with time. Our previous studies [48] showed that with a further distance from the membrane surface the theoretical models describe the evolution of the concentration field more correctly than for the membrane surface itself. The high values of *J*_s_ in a non-stationary state (0 ~ 20 min) indicate a high amount of transported substance, which causes an increase of concentration polarization and a rapid decrease of concentration difference on both sides of the membrane at the initial moment.

The strong influence of CBLs on the transport processes in the membrane system is confirmed by our previous studies of time evolution of CBLs [1, 43]. The particularly rapid increase of the CBL thickness occurs at the initial time points of their formation (0 < *t* < 500 s) and, over the same interval we observe a rapid decrease of the solute flux. The rapid decrease of the flux value also indicates that the diffusion flow of the solute through the membrane dominates over the free diffusion in the solution. In such a case, the solute accumulation occurs on one side and the solute depletion on another side of the membrane.

To illustrate directly the influence of CBLs on concentration polarization in the system, in Fig. 6 the *ζ*_s_ coefficient dependence on the CBL thickness is shown. This coefficient decreases systematically with the increase of thickness *δ* but for the range 0–1 mm, a particularly high decrease of *ζ*_s_ is observed.

Figure 7 presents the experiment and theoretical time dependencies of the relative permeability coefficient ζ_s_ as well as the dependence ζ_s_(*t*) calculated according to formula (3) on the basis of time evolution of the CBL thickness *δ*(*t*) obtained interferometrically. The theoretical dependencies ζ_s_(*t*) differ but all are in sufficient conformity with experimental data. The course of the theoretical curves indicates the full independence of this coefficient upon the solution concentration. Moreover, the experimental dependencies suggest that this coefficient for the used solution concentrations seems to be independent of (or weakly dependent on) the initial concentration of the solute. Both the theoretical and experimental results show that the relative permeability coefficient of the M/CBLs system decreases non-monotonically in time similar to the solute flux *J*_s_.

As already mentioned, the CBL can be treated as pseudo-membranes in series with the physical membrane. Ginzburg and Katchalsky [49] introduced a relation between the apparent permeability coefficient of the M/CBLs system (ω_s_), the true membrane permeability coefficient (ω_m_) and the solute permeability (CBL) coefficient (ω_*δ*_) of CBL:

13 $$ \frac{1}{\omega_s}=\frac{1}{\omega_m}+\frac{2}{\omega_{\delta }}. $$

Figure 8 shows the values of the ω_*δ*_ coefficient calculated from formula (13) on the basis of the relative permeability coefficient ζ_*s*_. The shapes of the ω_*δ*_(*t*) and *J*_s_(*t*) characteristics are similar and confirm that the transport processes in the system are determined mainly by the solute permeability of CBLs. The ω_*δ*_ coefficient value in the steady state is equal to about 0.15 × 10^−9^ mol/Ns and is approximately ten times lower than the value of the membrane permeability coefficient ω_m_.

A comparison of the two presented theoretical models shows that the first model provides a correct fit to the experimental data but it may not be sufficient to describe the biological membranes of a complex structure. This model contains only one parameter associated with the coefficients *k* and *D*_m,_ which describe the resultant effect of the membrane’s behavior. The second model is more complex and requires a precise determination of additional parameters (e.g., *D*_m_ in a series of separate experiments) but it gives a more detailed description of transport processes. Moreover, it contains the partition coefficient, which is frequently used in the description of the transport and characterizes the behavior of the membrane in relation to the dissolved substance and the solvent. In our opinion, to describe substance transport both models can be used but the first model (due to a simplified description) can be preferentially used for membranes with a simple structure, while the second model is more appropriate for biological membranes.

In conclusion, after a short time (after 15 ~ 20 min) in the membrane system a quasi-steady state is reached and strong concentration polarization occurs. The diffusive flow of the substance through the membrane prevails over the free diffusion of the substance in the solution and causes the accumulation of the substance around the membrane and creation of CBL. The value of the partition coefficient (*k* = 0.25) for the used substance and membrane indicates that accumulation of the diffusing substance does not occur in the membrane. The permeability of the M/CBL system is significantly lower than the permeability of the membrane. The relative permeability coefficient seems to be independent of or weakly dependent on the initial concentration of the solute. Concentration polarization significantly modifies many transport parameters and thereby affects the functioning of artificial systems as well as biological systems.

## References

[1] Ślęzak A, Dworecki K, Ślęzak IH, Wąsik S (2005). Permeability coefficient model equations of the complex: membrane-concentration boundary layers for ternary nonelectrolyte solutions. J. Membr. Sci..

[2] Bryll A, Michalska-Małecka K, Grzegorczyn S, Ślęzak A (2009). Model equation of relative solute permeability coefficient of membrane-concentration boundary layers complex. Polimery.

[3] Barry PH, Diamond JM (1984). Effects of unstirred layers on membrane phenomena. Physiol. Rev..

[4] Sistat P, Pourcelly G (1997). Chronopotentiometric response of an ion exchange membrane in the underlimiting current range. Transport phenomena within the diffusion layers. J. Membr. Sci..

[5] Pohl P, Saparov SM, Antonenko YN (1998). The size of the unstirred layer as a function of the solute diffusion coefficient. Biophys. J..

[6] Zydney AL (1997). Stagnant films model for concentration polarisation in membrane systems. J. Membr. Sci..

[7] Nikonenko VV, Lebedev KA, Suleimanov SS (2009). Influence of the convective term in the Nernst–Planck equation on properties of ion transport through a layer of solution or membrane. Russ. J. Electrochem..

[8] Kozmai AE, Nikonenko VV, Pismenskaya ND, Pryakhina OD, Sistat P, Pourcelly G (2010). Diffusion layer thickness in a membrane system as determined from voltammetric and chronopotentiometric data. Russ. J. Electrochem..

[9] Fernando WJN, Othman R (2006). Relevance of diffusion through bacterial spore coats/membranes and the associated concentration boundary layers in the initial lag phase of inactivation: a case study for *Bacillus subtilis* with ozone and monochloramine. Math. Biosci..

[10] Fernando WJN, Ahmad AL, Abd. Shukor SR, Lok YH (2008). A model for constant temperature drying rates of case hardened slices of papaya and garlic. J. Food Eng..

[11] Fernando WJN, Ahmad AL, Othman MR (2011). Convective drying rates of thermally blanched slices of potato (*Solamum tuberosum*): parameters for the estimation of drying rates. Food Bioprod. Process..

[12] Caputo M, Cametti C (2008). Diffusion with memory in two cases of biological interest. J. Theor. Biol..

[13] Yeap YY, Trevaskis NL, Porter CJH (2013). Lipid absorption triggers drug supersaturation at the intestinal unstirred water layer and promotes drug absorption from mixed micelles. Pharm. Res..

[14] Levitt MD, Strocchi D, Levitt G (1992). Human jejunum unstirred layer: evidence for extremely efficient luminal stirring. Am. J. Physiol..

[15] Fischbarg J, Li J, Kuang K, Echevarria M, Iserovich P (1993). Determination of volume and water permeability of plated cells from measurements of light scattering. Am. J. Physiol..

[16] Cotton CU, Reuss L (1989). Measurement of the effective thickness of the mucosal unstirred layer in Necturus gallbladder epithelium. J. Gen. Physiol..

[17] Winne D (1973). Unstirred layer, source of biased Michaelis constant in membrane transport. Biochim. Biophys. Acta.

[18] McLaughin SGA, Dilger JP (1980). Transport of protons across membranes by weak acids. Physiol. Rev..

[19] Peppenheimer JR (2001). Role of pre-epithelial “unstirred” layers in an absorption of nutrients from the human jejunum. J. Membr. Biol..

[20] Antonenko YN, Pohl P, Rosenfeld E (1996). Visualization of the reaction layer in the immediate. Arch. Biochem. Biophys..

[21] Schatz A, Reitstetter R, Briegleb W, Linke-Hommes A (1992). Gravity effects on biological systems. Adv. Space Res..

[22] Bizzarri M, Cucina A, Palombo A, Grazia Masiello M (2014). Gravity sensing by cells: mechanisms and theoretical grounds. Rend. Fis. Acc. Lincei.

[23] Todd P, Klaus DM (1996). Theories and models on the biology of cells in space. Adv. Space Res..

[24] Van Loon JJWA (2007). Micro-gravity and mechanomics. Gravit. Space Biol..

[25] Kordyum EL (2014). Plant gravisensitivity and adaptation to microgravity. Plant Biol..

[26] Tairbekov MG (1990). Positional homeostasis of cell and the problem of morphogenesis in gravity field. Usp. Sovrem. Biol..

[27] Horneck G, Klaus DM, Mancinelli RL (2010). Space microbiology. Microbiol. Mol. Biol. Rev..

[28] Mohan, V.P.C., Talukdar, P.: Three-dimensional numerical modeling of simultaneous heat and moisture transfer in a moist object subjected to convective drying. Int. J. Heat Mass Transfer **53**, 4638–4650 (2010)

[29] Hristov J (2011). Approximate solutions to fractional subdiffusion equations. Eur. Phys. J. Spec. Topics.

[30] Pedley TJ (1983). Calculation of unstirred layer thickness in membrane transport experiments. Q. Rev. Biophys..

[31] Abu-Rjal R, Chinaryan V, Bazant MZ, Rubinstein I, Zaltzman B (2014). Effect of concentration polarization on permselectivity. Phys. Rev. E.

[32] Schlichting H, Gersten K (2000). Boundary Layer Theory..

[33] Ślęzak A, Grzegorczyn S, Batko KM (2012). Resistance coefficients of polymer membrane with concentration polarization. Transp. Porous Media.

[34] Batko KM, Ślęzak-Prochazka I, Ślęzak A (2015). Network hybrid form of the Kedem–Katchalsky equations for non-homogenous binary non-electrolyte solutions: evaluation of *P*_*ij*_^*^ Peusner’s tensor coefficients. Transp. Porous Media.

[35] Wąsik S, Arabski M, Drulis-Kawa Z, Gubernator J (2013). Laser interferometry analysis of ciprofloxacin and ampicillin diffusion from liposomal solutions to water phase. Eur. Biophys. J..

[36] Wąsik S, Arabski M, Dworecki K, Janoska J, Semaniak J, Szary K, Ślęzak A (2014). Laser interferometric analysis of glucose and sucrose diffusion in agarose gel. Gen. Physiol. Biophys..

[37] Mattisson C, Roger P, Jönsson B, Axelsson A, Zacchi G (2000). Diffusion of lysozyme in gels and liquids. A general approach for the determination of diffusion coefficients using holographic laser interferometry. J. Chromatogr. B.

[38] Mattisson C, Karlsson D, Pettersson SG, Zacchi G, Axelsson A (2001). Light deflection and convection in diffusion experiments using holographic interferometry. J. Phys. D Appl. Phys..

[39] Roger P, Mattisson C, Axelsson A, Zacchi G (2000). Use of holographic laser interferometry to study the diffusion of polymers in gels. Biotechnol. Bioeng..

[40] Axelsson A, Marucci M (2008). The use of holographic interferometry and electron speckle pattern interferometry for diffusion measurement in biochemical and pharmaceutical engineering applications. Optic. Lasers Eng..

[41] Xiang TX, Xu YH, Anderson BD (1998). The barrier domain for solute permeation varies with lipid bilayer phase structure. J. Membr. Biol..

[42] Zocher F, van der Spoel D, Pohl P, Hub JS (2013). Local partition coefficients govern solute permeability of cholesterol-containing membranes. Biophys. J..

[43] Dworecki K, Kosztołowicz T, Wąsik S, Mrówczyński S (2000). Time evolution of near membrane layers. Eur. J. Phys. Educ..

[44] Hoogervorst CJP, de Goede J, Versluijs CW, Smit JAM (1978). Nonstationary diffusion through membranes. 2. Transient diffusion through a membrane separating two seminfinite volumes of unstirred solutions. J. Phys. Chem..

[45] Horcas I, Fernández R, Gómez-Rodríguez JM, Colchero J, Gómez-Herrero J, Baro AM (2007). WSXM: a software for scanning probe microscopy and a tool for nanotechnology. Rev. Sci. Instrum..

[46] Dworecki K, Wąsik S (1997). The investigation of time-dependent solute transport through horizontally situated membrane: the effect of configuration membrane system. J. Biol. Phys..

[47] Dworecki K, Ślęzak A, Drabik M, Ornal-Wąsik B, Wąsik S (2006). Determination of the membrane permeability coefficient under concentration polarisation conditions. Desalination.

[48] Dworecki K, Ślęzak A, Ornal-Wąsik B, Wąsik S (2005). Evolution of concentration field in a membrane system. J. Biochem. Biophys. Methods.

[49] Ginzburg BZ, Katchalsky A (1963). The frictional coefficients of the flows of nonelectrolytes through artificial membranes. J. Gen. Physiol..

